# Application of ZnO Nanoparticles for Improving the Thermal and pH Stability of Crude Cellulase Obtained from *Aspergillus fumigatus* AA001

**DOI:** 10.3389/fmicb.2016.00514

**Published:** 2016-04-18

**Authors:** Neha Srivastava, Manish Srivastava, P. K. Mishra, Pramod W. Ramteke

**Affiliations:** ^1^Department of Molecular and Cellular Engineering, Sam Higginbottom Institute of Agriculture Technology & SciencesAllahabad, India; ^2^Department of Chemical Engineering and Technology, Indian Institute of Technology (Banaras Hindu University)Varanasi, India; ^3^Department of Physics and Astrophysics, University of DelhiDelhi, India

**Keywords:** zinc oxide, nanoparticles, cellulase, *Aspergillus fumigatus*, thermal stability, pH stability, biofuels production

## Abstract

Cellulases are the enzymes which are responsible for the hydrolysis of cellulosic biomass. In this study thermal and pH stability of crude cellulase has been investigated in the presence of zinc oxide (ZnO) nanoparticles. We synthesized ZnO nanoparticle by sol-gel method and characterized through various techniques including, X-ray Diffraction, ultraviolet-visible spectroscope, field emission scanning electron microscope and high resolution scanning electron microscope. The crude thermostable cellulase has been obtained from the *Aspergillus fumigatus* AA001 and treated with ZnO nanoparticle which shows thermal stability at 65°C up to 10 h whereas it showed pH stability in the alkaline pH range and retained its 53% of relative activity at pH 10.5. These findings may be promising in the area of biofuels production.

## Introduction

Cellulases are the industrially important enzymes and offer wide range of applications in various fields such as detergent, beverages and biofuels industries (Li et al., 2014; Srivastava et al., 2015c). In the biofuels production process, cellulases play a crucial role in the hydrolysis of cellulosic biomass into the fermentable sugars. For the enzymatic hydrolysis of cellulosic biomass, cellulase system should have three main enzyme components namely, β-1,4-endoglucanase (EC 3.2.1.4), β-1,4-exoglucanase (EC 3.2.1.91), and β-glucosidase (EC 3.2.1.21) (Goyal et al., 1991; Hongxing et al., 2016; Srivastava and Jaiswal, 2016). Endoglucanase (EG) involves in breaking-down the crystalline structure of cellulose microfibrils to release separate polysaccharide chains whereas exoglucanase [cellobiohydrolases (CBH)], gradually alters long-chain cellulose into the cellodextrins; and β-glucosidase (BGL), converts cellodextrins into distinct glucose molecules. Thus, synergy actions of all these enzyme components are required for the effective hydrolysis of cellulosic biomass (Ang et al., 2013; Rawat et al., 2014b; Srivastava et al., 2015a). Moreover, the presently available enzymatic hydrolysis technologies for cellulosic biomass are generally carried out at 45–50°C, show slow rates of enzymatic hydrolysis, low yields of sugars, incomplete hydrolysis reaction, the need of high enzymes loading, and are very susceptible to the microbial contamination. These limitations could be resolved using the thermostable enzymes (Yeoman et al., 2010; Bhalla et al., 2013; Srivastava et al., 2015b).

Thermostable enzymes are stable at temperatures relatively higher as compared to the optimum temperature of the enzymes. An enzyme or protein can be regarded as the thermostable by nature if it retains its half-life at comparatively higher temperatures for a longer period (Liang et al., 2011). One of the important commercial applications of the thermostable cellulase includes fast enzymatic hydrolysis of cellulosic biomass into the fermentable sugars for biofuels production (Liang et al., 2011; Srivastava et al., 2014a). Beside, thermal stability, alkali-stability of cellulases is another important factor in the industries related to the biofuels as well as pulp bleaching process. Henceforth, cellulase enzyme having such types of functional characteristics like thermal and alkali stability have broad industrial applications. Fungi are the potential cellulase producer where the *Aspergillus fumigatus* is well known for the cellulase production (Ang et al., 2013; Srivastava et al., 2015b).

Nanomaterials can play a vital role to improve the thermal, and pH stability of the cellulase enzymes significantly due to the several unique physical and chemical properties (Pandurangan and Kim, 2015). Large surface to volume ratio, high surface reaction activity, high catalytic efficiency and strong adsorption ability are the important properties of nanomaterials which can improve the thermal and alkali stability of the cellulase enzymes (Willner et al., 2006; Ansari and Husain, 2012). Additionally, the large surface area of nanomaterials may contribute as the matrix for the immobilization of enzymes leading to increased stability. Furthermore, the multipoint attachment of the enzyme molecules on the surface of the nanomaterials can reduce the protein unfolding, resulting in the improved stability of the enzyme involved on the surface of nanoparticles (Kamyar et al., 2011; Zhu et al., 2014). In our earlier studies, we have shown the effect of NiCo_2_O_4_, Fe_3_O_4_ nanoparticles and Fe_3_O_4_/Alginate nanocomposite on the thermal stability of cellulase enzymes apart from its production and hydrolysis efficiency (Srivastava et al., 2014b, 2015b). Besides, improvement in stability, nanomaterial bound enzyme can also provide many advantages such as easy separation from the reaction mixture, less prone to contamination of product via the enzyme, continuous processing and the better product yield (Wenjuan et al., 2015). Among various types of nanomaterials, ZnO have the exceptional property of high biocompatibility, electron mobility and high isoelectric point (IEP ~ 9.5) which is suitable to enhance the long-term stability of the enzymes. Several groups have extensively studied the binding of ZnO nanomaterial and its consequence on the conformation of the protein (Li et al., 2005; Hu et al., 2007; Zhang et al., 2007). Consequently, ZnO nanoparticles can be an ideal material to study the improvement in the catalytic efficiency of the enzymes.

Therefore, in the present work, we synthesized ZnO nanoparticles by sol-gel method and characterized by several techniques. Furthermore, an attempt has been made to evaluate the effect of ZnO nanoparticles on the thermal and pH stability of cellulase enzyme.

## Materials and Methods

### Materials

Zinc acetate dihydrate [Zn(CH_3_COO)_2_⋅2H_2_O], ammonium hydroxide solution [NH_4_OH], glycolic acid [C_2_H_4_O_3_] and deionized water have been used in this experimental process. All the chemicals used in the entire experiment were of analytical grade and further used without any purification unless otherwise stated.

All the analytical chemicals, media components and reagents used in this study were procured from the Himedia laboratories (Mumbai, India), Titan Biotech, CDH and Fisher Scientific (Mumbai, India).

### Feed Stocks Used for the Enzyme Production

Rice straw of variety AS-116 was procured from the research fields of Allahabad, Uttar Pradesh, India. Wheat bran was purchased from the local floor mill. Rice straw was dried, cut into small pieces and ground into particles size of around 2 mm whereas wheat bran was used as such for the enzyme production.

### Preparation of ZnO Nanoparticiples

In this method, we have synthesized ZnO nanoparticiples by sol-gel method (Srivastava et al., 2010; Ojha et al., 2014). The following steps were carried out; firstly, 0.2 mol solution of zinc acetate dihydrate was prepared using the deionized water. After that, glycolic acid was added into the above prepared solution with a rigorous stirring. The molar ratio of metal salts to glycolic acid was kept to 1:2.5. Subsequently, ammonium hydroxide solution was slowly added in this solution to maintain the pH of the solution ~ 10. After stirring up to 2 h, the solution was kept in the oven for the evaporation of extra amount of water at temperature 60°C. Thereafter, the obtained solution was again heated at 70°C with vigorous magnetic stirring which yield a white slurry type material. Finally this slurry type material was calcined at 500°C for 6h to obtain the final product which was further crushed into the fine powder and characterized by several techniques.

### Characterizations of ZnO Nanoparticiples

The synthesized product was characterized through X-ray diffraction (XRD) (Philips X’Pert Pro-diffractometer), equipped with nickel filter and copper target. The wavelength of X-ray used in this study was 1.54060 nm. The Ultraviolet–visible (UV-Vis) spectrum (Hitachi-330 spectrometer) was recorded in the wavelength range of 200–800 nm. The surface morphology of the synthesized product was investigated through the field emission scanning electron microscope (FE-SEM) (SIRIDN-2000). On the other hand shape and size of the particles were measured through the high-resolution transmission electron microscope (HR-TEM) (Tecnai-F30).

### Isolation and Screening of the Fungal Isolates

The isolation of cellulase producing fungal strain have been done from the rotten wood sample, collected from the local area of Allahabad, India, using the serial dilution technique (Srivastava et al., 2015b). The detailed procedure of isolation and screening has been provided in the Supplementary File (see Isolation and Screening of the Fungal Isolates).

### Molecular Identification of the Screened Fungal Isolate

Identification study of the selected fungal strain has been performed using 18S region based molecular technique, and detail information of the process has been explained in the Supplementary File (see Molecular Identification of the Screened Fungal Isolate) (Tamura et al., 2007; Srivastava et al., 2015b).

### Enzyme Production

Solid state fermentation (SSF) has been performed for the production of cellulase enzyme and procedure of the process has been discussed in the Supplementary File (see Enzyme Production) (Srivastava et al., 2015b).

### Activity Assay of Cellulase

Filter paper cellulase (FP)/Endoglucanase and β-glucosidase activity were investigated by following the method of Kubicek (1982) and Ghose (1987) respectively, while reducing sugar concentration was confirmed using the dinitrosalicylic acid (DNS) method (Miller, 1959) and protein content was estimated by Bradford (1976) method. Details regarding all these procedures have been given in the Supplementary File (see Activity Assay of Cellulase).

### Thermal Stability of the Cellulases in the Presence of ZnO Nanoparticles

Based on our earlier investigation and keeping the application of the study in focus, the present experimental studies have been performed on the basis of FP activity (Srivastava et al., 2015b). As discussed in the previous studies, it is the measurement of the overall activity of cellulase enzyme complex required for the hydrolysis of cellulosic substrates (Teeri, 1997; Rawat et al., 2014a; Srivastava et al., 2015b). Since the crude cellulase was observed to be thermally stable for 4 h at 60°C, further studies on thermal stability of ZnO nanoparticles treated cellulase was done in the presence of different concentrations (2.5–15 μg/mL) of ZnO nanoparticles at 60°C for 4 h. In addition, based on the obtained results, we decided to perform the thermal stability experiment at the selected concentration of 7.5 μg/mL of ZnO nanoparticles treated cellulase for 5–12 h at different temperature range from 60 to 100°C. Immediate enzyme assay for residual activity was performed and reflected as relative to the enzyme activity of control cellulase.

### pH Stability of Cellulases in the Presence of ZnO Nanoparticles

The pH stability of the crude cellulase in the presence of ZnO nanoparticles has been carried out under the same experimental conditions and the selected concentration of ZnO nanoparticles at pH range of 7.5–12 for 60 min at 60°C. The 0.1 M Tris-HCl was used for the pH range 7.5–10.0 and 0.1 M glycine-sodium hydroxide was used for the pH 11–12. Thereafter, the relative activities are determined by the ratio of the enzyme activity of each sample and the maximum activity of all samples.

### Statistical Analysis

All the experiments were done in triplicate, and the means and standard deviation (SD) values were calculated using the excel program. The data was analysis using variance (ANOVA), SPSS (version 16). Further, Turkey’s test was used to test the significance of the difference between the treatment means.

## Results and Discussion

### XRD and UV-Vis Studies

**FIGURE 1:**
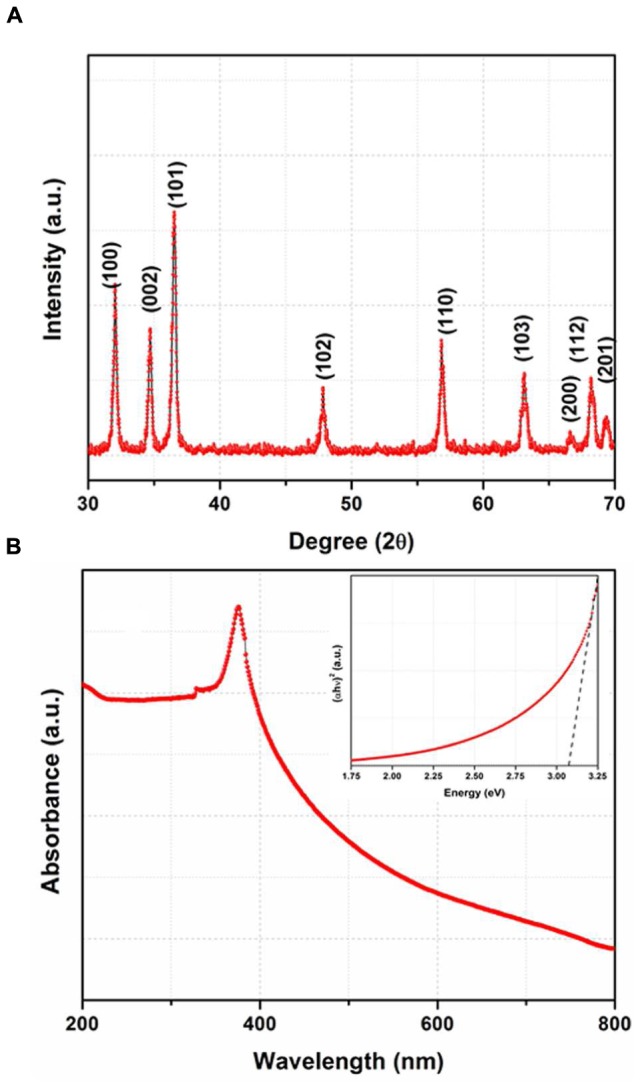
**(A)** X-ray Diffraction (XRD) pattern of the synthesized product, **(B)** UV-Vis spectrum of the ZnO nanoparticles and inset shows Tauc plot.

The XRD pattern of the synthesized product is shown in **Figure 1A**. The XRD pattern exhibits nine well defined diffraction peaks and all the peaks are indexed with the help of joint committee on powder diffraction standards (JCPDS card no. 36-1451) (Deng et al., 2016). These peaks are corresponding to (100), (002), (101), (102), (110), (103), (200), (112), and (201) planes which suggest the formation of wurtzite type crystal structure. Besides, no peak corresponding to any impurity could be observed and confirm the formation of single phase ZnO.

The UV-Vis spectrum of ZnO nanoparticles is shown in **Figure 1B** which shows an absorption peak ~ 365 nm. As shown in the inset of (**Figure 1B**), the optical energy band gap is determined by plotting the graph between hυ vs. (αhυ)^2^ where the linear extrapolation of the plots gives the value of band gap. The value of band gap is calculated to be ~ 3.15 eV. This value of band gap is consistent with the value reported in earlier studies (Ozgur et al., 2005; Cui et al., 2016; Klubnuan et al., 2016; Pathak et al., 2016; Wang et al., 2016).

### FE-SEM and HR-TEM Studies

**FIGURE 2:**
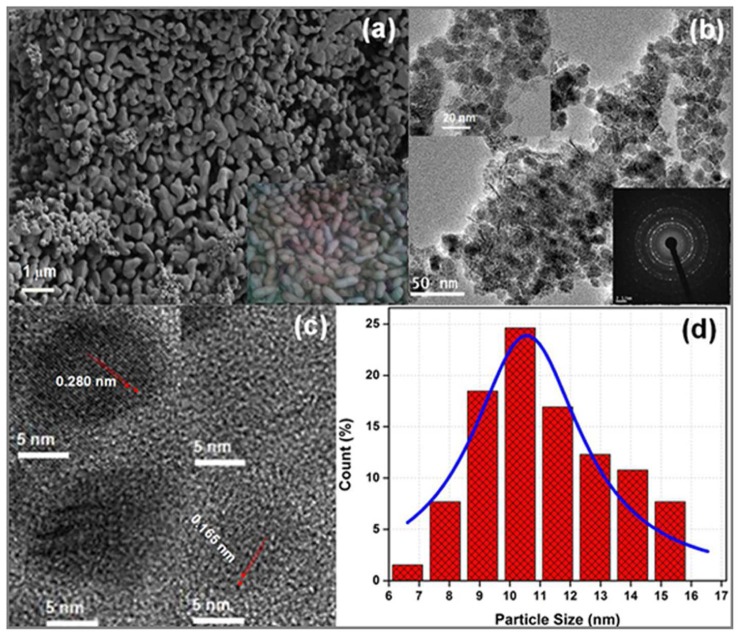
**(a)** Field emission scanning electron microscope (FE-SEM) image (inset shows snap of real peanuts), **(b)** TEM (upper inset shows magnified image and lower inset shows SAED pattern), **(c)** HR-TEM micrograph and **(d)** histogram of ZnO nanopaticles fitted by Lorentzian function.

**Figure 2a** represents FE-SEM micrograph of the ZnO nanoparticles. It can be seen that the surface morphology of ZnO resembles peanut types structure, and the particles are uniformly distributed over the entire micrograph. The grain size can be measured in the range of 0.1–1.0 μm. To observe the actual size and shape of the ZnO nanoparticles, HR-TEM micrographs are shown in **Figures 2b,c**. The HR-TEM micrographs show that particles are nearly spherical in shape and their size lies between 6 and 17 nm. The selected area electron diffraction pattern (SAED) (lower inset of **Figure 2b**) shows series of rings which define the polycrystalline nature of ZnO nanoparticles (Srivastava et al., 2013). Moreover, as presented in **Figure 2c**, the HR-TEM image suggest that the particles are well crystallized where the lattice fringes are clearly observed. The interplanar spacing of 0.280 nm, and 0.165 nm corresponding to (100) and (101) are in consistent with the XRD results. The quantitative measurement of the size of the particles is also done by plotting the histogram and fitted by Lorentzian function (**Figure 2d**). The average size of the particles from the histogram is measured to be 12.56 ± 0.26 nm (Srivastava et al., 2013).

### Identification of Selected Fungal Isolate

The selected fungal isolate has been identified as *A. fumigatus* AA001 using the molecular characterization technique of 18S ribosomal DNA (rDNA). The sequence of the *A. fumigatus* AA001 has been deposited in NCBI and accession no. KM592797 has been allotted (Supplementary Figure S1). The details of identification about the selected strain have been given in the Supplementary File (see Identification of Selected Fungal Isolate) (Srivastava et al., 2015b).

### Effect of ZnO Nanoparticles on the Thermal Stability of Crude Cellulases

**FIGURE 3:**
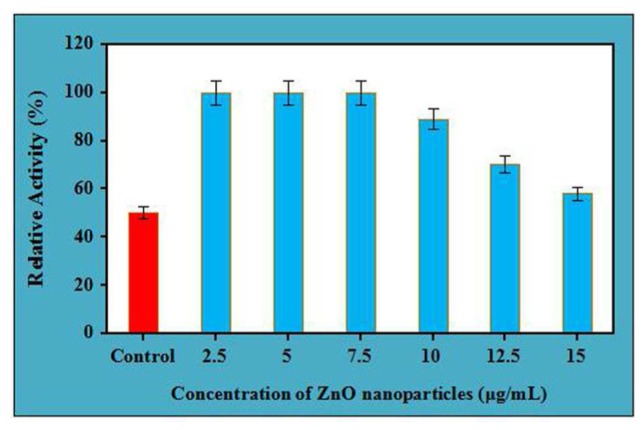
Thermal stability of crude cellulase in presence of different concentration of ZnO nanoparticles at 60°C.

Thermal stability of crude cellulase was performed at different concentrations of ZnO nanoparticles for 4 h. It is clear from the (**Figure 3**), at 60°C, enzyme showed its 100% stability at three different concentrations. It was recorded that at the same temperature, the enzyme reflected its maximum stability at concentration 7.5 μg/mL for 4 h whereas it retains its 89 and 70% relative activity at the concentrations of 10 μg/mL and 12.5 μg/mL, respectively (with a significance level of 1.0%). However, the enzyme retained its 58% of relative activity for 4 h at the concentration of 15 μg/mL, which shows fall in the enzyme activity beyond the concentration of 7.5 μg/mL for the same period. It was striking to mention here that the untreated cellulase which was designated as the control retained its half-life for 4 h while ZnO nanoparticles treated cellulase retained its 100% stability for the same period between the concentration of 2.5 μg/mL to 7.5 μg/mL. Moreover, it is well documented that the immobilization property of nanoparticles improves the stability of enzyme, and the biocompatibility of ZnO nanoparticles might be a reason to retain the complete stability of ZnO nanoparticles treated cellulase as compared to the control at the same temperature and time. However, decreasing the stability of ZnO nanoparticles treated cellulase on increasing the concentration over 7.5 μg/mL might be due to the non-supportive interaction of nanoparticles with the enzyme at higher concentrations.

According to the study of Lynch et al. (2007) and Monopoli et al. (2012), when protein is treated with nanoparticles, the concern nanoparticles try to interact, adsorb proteins continuously and form a structure known as protein “corona” which dramatically changes their surface properties. Additionally, the corona formed from the successful interaction of nanoparticles, and protein is completely different from the corona which is developed when the same nanoparticle was present in bulk with protein under the same experimental conditions. Thus, the amount of nanoparticles is most likely that would influence its interaction and stability with protein, and this might be the very possible reason for decrease in stability of cellulase on increasing the concentration of ZnO nanoparticles. In spite of this, there are many other factors which affect the interaction of the protein with nanoparticles like the physiochemical properties of the nanoparticles and the composition of the protein, results in feasible reversible/irreversible conformational changes in the structure of the protein with nanoparticles (Chakraborti et al., 2013). Moreover, the celulase enzyme, used in the present study was thermostable by nature while the ZnO nanoparticles have a high isoelectric point, therefore capable of better immobilization property which might be helpful for generating better corona to improve the stability.

**FIGURE 4:**
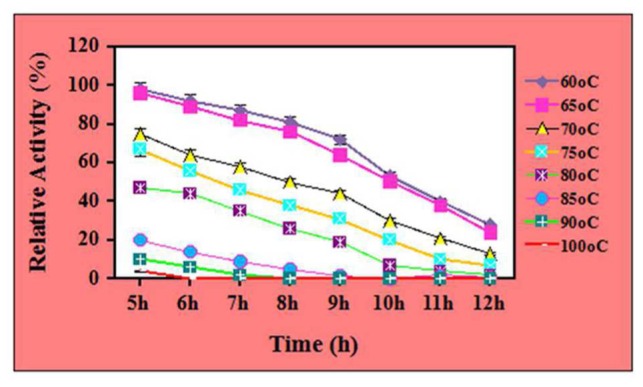
Thermal stability of ZnO nanoparticles (7.5 μg/mL) treated cellulase at different temperatures.

Further, the thermal stability of 7.5 μg/mL ZnO nanoparticles treated cellulases was tested for 5–12 h at different temperature ranges from 60 to 100°C. The results showed that ZnO nanoparticles treated cellulase showed its half-life for 10 h at 65°C, whereas the same retained ~76% and 64% of relative activity for 8 and 9 h, respectively. However, it maintained ~96, 89, and 82 for 5, 6, and 7 h of incubation (with significance level of 1.0%) (**Figure 4**). However, the relative activity of the enzyme was observed to be decrease and it retained ~38 and 24% for 11 and 12 h. In contrast, the nanoparticle-treated cellulase retained its 56, 46, and 38% of relative activity at 75°C after the incubation of 6, 7, and 8 h, respectively. Additionally, the same owned 31, 20, 10, and 7% stability after the incubation of 9, 10, 11, and 12 h, respectively. Nevertheless, at 100°C, the ZnO nanoparticles treated cellulase reflects its 4 and 0% of relative activity after 5 and 6 h of incubation (with the significance level of 1.0%). These results clearly indicate the potential of ZnO nanoparticles to improve the stability of crude cellulase at higher temperatures for longer duration. When compare to control, the ZnO nanoparticles treated cellulase showed 2.5 times better stability. The biocompatibility and the isoelectric point could the most appropriate reason to elucidate this phenomenon. Biocompatibility is a well-known concept to improve the stability of the enzyme. Apart from the biocompatibility, other fascinating properties of ZnO nanoparticles such as wide band gap, high exciting binding energy, and quantum effects may also support to make it an ideal candidate for improving the stability of cellulase enzyme. Moreover, since the hexagonal wurtzite structure of ZnO nanoparticles is thermodynamically stable, it may be helpful to influence the thermal stability of crude cellulase enzymes (Ashrafi and Jagadish, 2007; Anders et al., 2015). Thus, use of such type of thermally stable ZnO nanoparticles treated cellulase can improve the economics of market involved in the enzyme-based hydrolysis of biomass, because the enzymatic degradation of cellulose needs the high enzyme loading which increases the production cost as well as economic demands. Since the ZnO nanoparticles treated cellulase are thermally stable for longer duration, so enzymatic bioconversion related issues like incomplete hydrolysis, high enzyme loading, low yield can be avoided. Besides, biofuels, the ZnO-treated cellulase may also serve for the industries involved in the fibers processing and textiles that require reactions at elevated temperatures for long duration (Vyas and Lachke, 2003; Srivastava et al., 2015b).

The effects of nanomaterials on the thermal stability of enzyme have been reported in some of the studies. In one of the studies by Cherian et al. (2015), the cellulase immobilized on MnO_2_ nanoparticles showed thermal stability for 2 h while the control (without immobilization) showed its thermal stability for 1 h, only. In one of the other study by Zhang et al. (2015), the cellulase immobilized at functionalized magnetic nanoparticles showed thermal stability about 97.8% at 40°C. Kamyar et al. (2011), reported the thermal stability of cellulase immobilized on superparamagnetic nanoparticles for 3 h at 60°C. In our earlier studies, we have reported the half-life of crude cellulase in the presence of NiCo_2_O_4_ nanoparticles for 7 h at 80°C (Srivastava et al., 2014b) and ~56% at 70°C for 8 h in the presence of Fe_3_O_4_/Alginate nanocomposite (Srivastava et al., 2015b). Our results are in agreement with the report of Husain et al. (2011). These authors have reported that the ZnO nanoparticles immobilized β-glucosidase exhibited 55% of relative activity for 2 h at 60°C. These results suggest the industrial potential of ZnO nanoparticles treated cellulase are not limited in the area of biofuels production, only.

### Effect of ZnO Nanoparticles on pH Stability of Crude Cellulases

**FIGURE 5:**
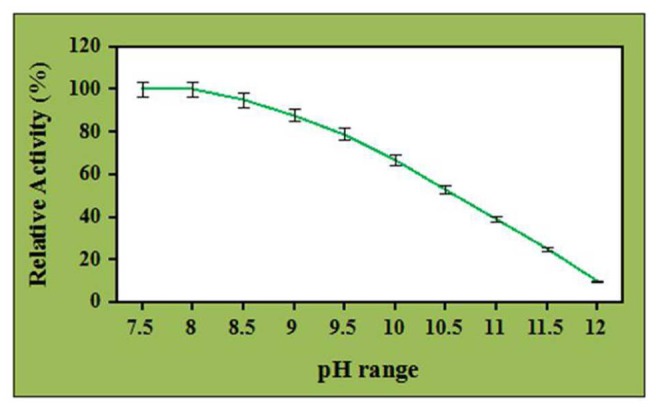
Effect of ZnO nanoparticles (7.5 μg/mL) on pH stability of crude cellulase.

Effect of ZnO nanoparticles on the pH stability of the crude cellulase has been investigated at different pH ranges and depicted in **Figure 5**. It was observed that the enzyme was stable and retained its complete relative activity at pH 7.5 which was continued to stable in the presence of ZnO nanoparticles at pH 8.0. Further, the pH stability of ZnO nanoparticles treated cellulase was decreased with increase in the pH value and it retained its 67 and 53% of relative activity at pH 10 and 10.5. However, the enzyme retained its 39 and 10% of relative activity at pH 11 and 12 (with significance level of 1.0%). Nevertheless, when compared to control, the ZnO nanoparticles treated cellulase showed better performance by maintaining its half-life at pH 10.5 whereas it was observed that the untreated cellulase retained its 52 and 32% of relative activity at pH 8.0 and 8.5, respectively. Additionally, the untreated cellulase retained non-considerable relative activity beyond the pH 8.5 (data not shown). These results clearly indicated the potential role of ZnO nanoparticles to improve the pH stability and efficiency of crude cellulase enzyme. In addition, ZnO nanoparticles having a high isoelectric point (IEP ~9.5) is suitable for the adsorption of low IEP enzymes such as cellulase (IEP ~4.5) and this could probably be a reason for the improved alkali stability of ZnO nanoparticles treated celluase by providing a friendly micro-environment to retain its stability (Wang et al., 2013).

The effect of pH on the cellulase activity has been broadly studied. The impact of pH significantly depends on the fungal species. Nevertheless, very limited information is available about the fungal cellulases demonstrating pH stability in the alkaline range. The crude cellulase obtained from the *A. fumigatus* AA001 was alkali tolerant and this property was further improved in the presence of ZnO nanoparticles. Besides, this improved alkali tolerant crude enzyme has wide application in the biofuels production from the agriculture residues. Further, during the acidic or alkali treatment of cellulosic biomass, little amount of water is required to neutralize the pH of the biomass for enzymatic hydrolysis via this ZnO nanoparticles treated cellulase which may reduce the production cost up to a great extent.

In one of the studies by Verma et al. (2013), the pH stability of β-glucosidase enzyme from *A. niger* was observed at pH 6.0 in the presence of magnetic nanoparticles. In a very recent study by Zhang et al. (2015), the pH stability of cellulase was reported at pH 5.0 using the magnetic nanoparticles. Agrawal et al. (2016) have reported 84% of pH stability of β-glucosidase at pH 7.0. In the study by Husain et al. (2011), the pH stability of β-glucosidase from *A. oryzae* was observed at pH 4.5 using the ZnO nanoparticles. However, in the present study the crude cellulase obtained from the *A. fumigatus* AA001 was stable at pH 10.5 in the presence of ZnO nanoparticles. It is noteworthy to mention that, we are yet to come across any literature reporting the pH stability of the nanomaterial treated cellulase in the alkaline pH range. Also, it has been reported that apart from the fungal species, the activity and stability of an enzyme is chiefly based on the type of functional groups present therein which support to shift in the pH range (Takagi et al., 1977; Jones et al., 1981; Zhu et al., 2014). Additionally, high biocompatible nature of ZnO nanoparticles might be helpful to improve the conformational flexibility of enzyme as well as prevent the immobilized protein from the unfolding and denaturing (Mateo et al., 2000; Wang et al., 2009). Therefore, the long duration thermal stability and tolerance toward the high alkaline pH range makes this nanoparticles treated crude enzyme cocktail as an ideal candidate for efficient hydrolysis of cellulosic biomass and other related fields. Besides, the economy involved in the production of biofuels can also be improved because of the replacement of commercial cellulase with nanoparticle-treated cellulase. Though till date some reports are available about the enhancement of enzymatic activity and stability of cellulase and other industrial enzymes in the presence of nanomaterials, it needs further investigation from the bench to top scale to ensure its potential utility.

## Conclusion

In this study, an attempt has been made to report the improved thermal and pH stability of crude cellulase from the *A. fumigatus* AA001 using the ZnO nanoparticles. The crude enzyme system showed thermal stability at 65°C for 10 h and maximum pH stability at the alkaline value of pH 10.5. The most suited application of this work can be in the area of bioethanol and biohydrogen production for the bioconversion of cellulosic waste. These findings have the potential to develop an eco-friendly method for the efficient treatment of these kinds of waste and generation of energy as well.

## Author Contributions

NS performed the application part and written the manuscript, MS synthesized nanoparticles, characterized, analyzed the experimental results and edited the manuscript. PKM and PWR discussed the experimental results and edited the manuscript.

## Supplementary Material

The Supplementary Material for this article can be found online at: http://journal.frontiersin.org/article/10.3389/fmicb.2016.00514

## Conflict of Interest Statement

The authors declare that the research was conducted in the absence of any commercial or financial relationships that could be construed as a potential conflict of interest.
