# Supplementary material for: Application of ZnO Nanoparticles for Improving the Thermal and pH Stability of Crude Cellulase Obtained from Aspergillus fumigatus AA001
Source: Front Microbiol. 2016 Apr 18;7:514. doi: 10.3389/fmicb.2016.00514 (PMC4834293; doi:10.3389/fmicb.2016.00514)
Supplement: Supplementary file 1 [file Presentation_1.PDF]

frontiers

Supplementary Material

Application of ZnO nanoparticles for improving the thermal and pH stability of crude cellulase obtained from *Aspergillus fumigatus* AA001

Neha Srivastava[1,2], Manish Srivastava[3], P. K. Mishra[1] and , Pramod W. Ramteke[1]*

[1]*Department of Molecular and Cellular Engineering, Sam Higginbottom Institute of Agriculture Technology & Sciences (Formerly Allahabad Agricultural Institute) Deemed to be University, Allahabad 221007, Uttar Pradesh, India*

[2]*Department of Chemical Engineering and Technology, Indian Institute of Technology (Banaras Hindu University) Varanasi, India, 221005*

[3]*Department of Physics & Astrophysics, University of Delhi, Delhi 110007, India.*

* Corresponding authors
E-mail: pwramteke@gmail.com
pramod.ramteke@shiats.edu.in

Supplementary Material

1. Isolation and screening of the fungal isolates

For the isolation of cellulase producing fungal strains, sample of rotten wood have been collected from Allahabad, India in the month of May to June. The sample was transferred into 250-mL flask having 50 mL sterile distilled water and was mixed robustly via vortexed shaker around 15 min under aseptic condition. Further, the fungal strains were isolated via serial dilution method using sterilized plates of Rose-Bengal Chloramphenicol (RBC) and potato dextrose agar (PDA) medium. The plates were kept under regular observation for colony development upto four to six days at 45ºC. Depending on the morphological characteristic features such as colour of spores and colony characteristics, the different colonies were subculture on PDA plates in order to get pure fungal isolates. Stock cultures preservation was done at 4ºC by preparing PDA slants and glycerol stocks for identification and characterization studies and subcultures were maintained at the interval of 30 days for the active viability of fungal strain. Additionally, inoculum of each fungal isolate slants was plated on carboxymethycellulose (CMC) agar medium and incubated at 45ºC for 48 h. Afterwards, the incubated plates were flooded with Gram's iodine solution for 5 min. Cellulase production by various selected fungal strains was specified by the formation of a zone of clearance around the fungal colony (Kasana et al., 2008) and cellulolytic index (CI) was calculated and shown as the ratio between the diameter of the degradation halo as well as the diameter of the fungal colony (Srivastava et al., 2015).

1.1 Molecular identification of the screened fungal isolate

Identification study of the selected fungal strain has been performed using molecular identification technique (Srivastava et al., 2015). Genomic DNA extract and a template for PCR amplification of the 18S rRNA gene were used for this purpose. Products obtained from

PCR amplification were purified and exposed for sequence analysis. After that, the similarity search for selected sequence was done through BLAST program of NCBI (National Centre of Biotechnology Information) and phylogenetic analysis was done by neighbour-joining method via MEGA software programme (Tamura et al., 2007).

2. Enzyme production

For cellulase enzyme production, solid state fermentation (SSF) was performed in 250 ml flasks using rice straw (RS) and wheat bran (WB) in the ratio of 5:1 with Mandel weber medium (Mandels et al., 1969). Mineral solution of one litre have constituents; KH_2PO_4 (1.5 gL^{-1}), $MgSO_4.7H_2O$ (0.3 gL^{-1}), $CaCl_2.2H_2O$ (0.4 gL^{-1}), $(NH_4)_2SO_4$ (1.3 gL^{-1}), $FeSO_4.7H_2O$ (6.0 mgL^{-1}), $MnSO_4.H_2O$ (1.6 mgL^{-1}), $ZnSO_4.7H_2O$ (1.3 mgL^{-1}), $CoCl_2.6H_2O$ (2.0 mgL^{-1}), Peptone (0.8 gL^{-1}) and 2% Tween-20. Moisture content of 70% was made up using the Mandel weber medium. Initially, pH of the medium was adjusted to 8.0. Next, 10 % of spore suspension containing 10^7spores/ml was used as inoculum and after inoculation the flasks were incubated at 45°C for 120 h. After the completion of incubation period, flasks were kept on shaking with addition of buffer till 30 min followed by the centrifugation of the same culture suspensions at 5000 rpm for 8 min at 4°C. The gained supernatant after the centrifugation process was directly used for enzyme assays (Srivastava et al., 2015).

2.1 Activity assay of cellulase

Filter paper cellulase (FP)/Endoglucanase (EG) activity were done by the method of Ghose et al., (1987) whereas β-glucosidase activity was performed by following the method of Kubicek et al., (1982). Further, reducing sugar concentration was confirmed using the dinitrosalicylic acid (DNS) method (Miller et al., 1959). One unit (IU) of FP or EG activity was well-defined as the quantity of enzyme necessary to release one µmole of glucose per mL

per min from the substrate filter paper and CMC, respectively following the standard assay conditions. Next, one unit of β-glucosidase was defined as the amount of enzyme releasing 1 μmol of p-nitrophenol (pNP) per mL per min from p-nitrophenylglucopyranoside (pNPG). Specific activity of the enzyme was calculated as the concentration of the concern enzyme in the protein, reported as IU per mg protein. Protein concentration was evaluated via Bradford (1976) method using the bovine serum albumin (BSA) as a standard (Srivastava et al., 2015; Srivastava et al., 2014).

3. Identification of selected fungal isolate

Among the four selected isolates based on CI index, the isolate AA001 produced higher enzyme activities than other selected strains, and thus was selected for next studies. In addition, the 18S ribosomal DNA (rDNA) technique had confirmed the identification of the isolate AA001 via molecular characterization technique. Furthher, the phylogenetic analysis which is depended on BLAST search by applying 18S rDNA sequence revealed that the strain demonstrated supreme homology (100 %) with the fungus *Aspergillus fumigatus* strains 1819 with accession no. HQ871898.1 of the NCBI library [Fig S1]. Therefore based on the cladistics analysis as well as homology assessment, it was certain that the selected isolate which was tagged as AA001 could be regarded as *Aspergillus fumigatus* AA001 and the sequence of the same has been submitted in NCBI along with accession no. KM592797 (Srivastava et al. 2015).

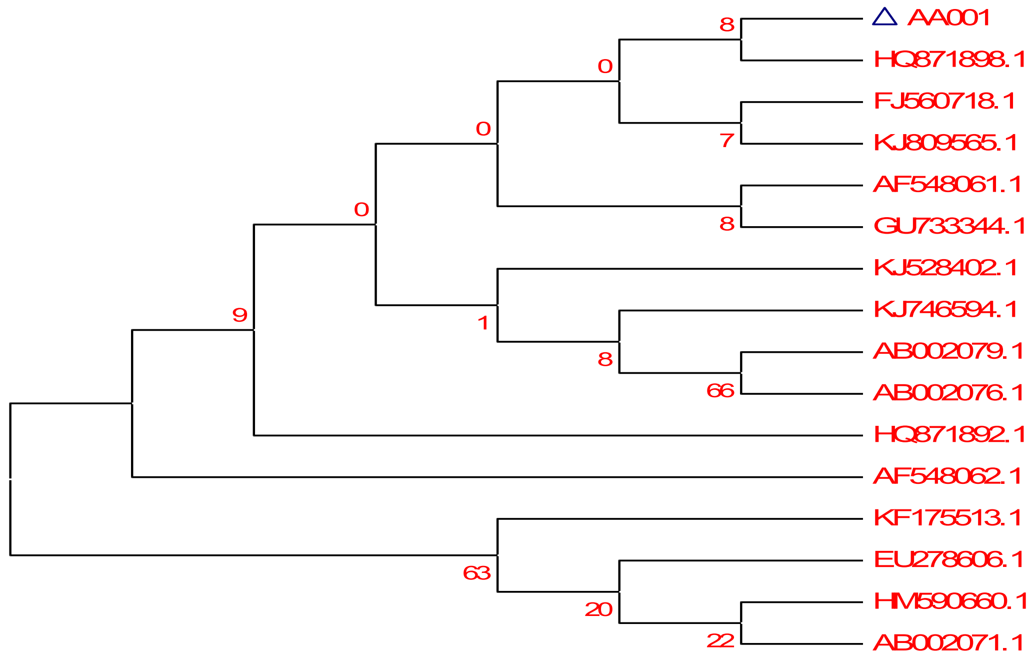

Fig. S1: Phylogenetic analysis of *Aspergillus fumigatus* AA001 [adopted with permission, Srivastava et al., 2015].

References

Ghosh, T. (1987). Measurement of cellulase activities. *Pure Appl. Chem.* 59, 257–268.

Kasana, R. C., Salwan, R., Dhar, H., Dutt, S., & Gulati, A. (2008). A rapid and easy method for the detection of microbial cellulases on agar plates using Gram's Iodine. *Curr. Microbiol*. 57, 503–507.

Kubicek, C. P. (1982). β-glucosidase excretion by *Trichoderma pseudokoningii*: correlation with cell wall bound β -1,3-glucanase activities. *Archives Microbiol.* 132, 349–354.

Mandels, M., Weber, J. (1969). The production of cellulases. *Adv. Chem.* 95, 391–414.

Marion, M. Bradford. (1976). A Rapid and Sensitive Method for the Quantitation of Microgram Quantities of Protein Utilizing the Principle of Protein-Dye Binding. Anal. Biochem. 72, 248-254.

Miller, G. L. (1959). Use of dinitrosalicylic acid reagent for determination of reducing sugar. *Anal. Chem.* 31, 426-428.

Srivastava, N., Rawat, R., Sharma, R., Oberoi, H.S., Srivastava, M., and Singh, J. (2014) Effect of Nickel–Cobaltite Nanoparticles on Production and Thermostability of Cellulases from Newly Isolated Thermotolerant *Aspergillus fumigatus* NS (Class: Eurotiomycetes). *Appl. Biochem. Biotechnol.* 174, 1092–1103.

Srivastava, N., Singh, J., Srivastava, M., Ramteke, P.W., and Mishra, P.K. (2015) Improved production of reducing sugars fromrice strawusing crude cellulase activated with Fe_3O_4/Alginate nanocomposite. *Bioresour. Technol.*, 183, 262–266

Tamura, K., Dudley, J., Nei, M., Kumar, S. (2007). MEGA4: Molecular Evolutionary Genetics Analysis (MEGA) software version 5.0. *Mol Biol Evol.* 24, 1596-1599.
